# Validation of the Japanese version of the Clinical Frailty Scale

**DOI:** 10.1111/ggi.15092

**Published:** 2025-02-02

**Authors:** Hitoshi Komiya, Yusuke Suzuki, Kazuhisa Watanabe, Masaaki Nagae, Hirotaka Nakashima, Chisato Fujisawa, Shuzo Miyahara, Tomihiko Tajima, Tomomichi Sakai, Yasushi Takeya, Taro Kojima, Yumi Umeda‐Kameyama, Shuji Kawashima, Hiroyuki Umegaki

**Affiliations:** ^1^ Centre for Community Liaison and Patient Consultations Nagoya University Hospital Nagoya Japan; ^2^ Department of Community Healthcare and Geriatrics Nagoya University Graduate School of Medicine Aichi Japan; ^3^ Division of Health Sciences Osaka University Graduate School of Medicine Osaka Japan; ^4^ Department of Geriatric Medicine The University of Tokyo Tokyo Japan; ^5^ National Center for Geriatrics and Gerontology Obu Japan

**Keywords:** clinical frailty scale, frailty, Japanese language, validation studies

## Abstract

**Aim:**

Among the many screening tools developed to detect frailty and disability in older adults, the Clinical Frailty Scale (CFS) is a valid, reliable, and easy‐to‐use tool and has been translated into several languages. The aim of this study was to demonstrate the validity of the Japanese version of the CFS (CFS‐J).

**Methods:**

The necessary permissions were obtained from Rockwood and colleagues. The Japanese version was prepared by translation (English to Japanese) and verified by back translation (Japanese to English). Concurrent criterion validity was assessed by evaluating the extent to which CFS relates to the Frailty Index (FI), the Frailty Index based on a Comprehensive Geriatric Assessment (FI‐CGA), and Performance Status (PS), using Kendall's tau.

**Results:**

In total, 223 patients admitted to the geriatric ward of an acute hospital between June 2021 and March 2023 were analyzed in the validation study. Mean age was 84.9 ± 5.9 years (range, 67–101 years), 128 (57.4%) were women, and the mean body mass index was 20.9 ± 4.0 kg/m^2^. In a correlation analysis, significantly positive associations were found between the CFS‐J and the FI (*r* = 0.713, *P* < 0.001), the FI‐CGA (*r* = 0.691, *P* < 0.001), and PS (*r* = 0.669, P < 0.001) for numerical variables. Significant positive associations were also found between the CFS‐J and the FI (*r* = 0.567, *P* < 0.001) and the FI‐CGA (*r* = 0.591, *P* < 0.001) for categorical valuables.

**Conclusion:**

The CFS‐J was found to be a valid instrument for identifying frailty in Japanese inpatients. **Geriatr Gerontol Int 2025; 25: 411–417**.

## Graphical abstract

This study was conducted to examine the validity of the Japanese version of the clinical frailty scale (CFS‐J) for assessing frailty and disability; the CFS‐J was found to be a valid tool for assessing frailty and disability.

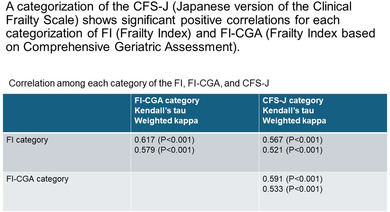

## Introduction

Frailty is a condition in which a person is prone to health problems due to various age‐related functional changes and loss of physiological reserve capacity,^1^ and it is associated with an increased risk of disability, falls, fractures, hospital admissions, institutionalization, and death.^2^ Therefore, it is important to assess frailty and provide appropriate interventions in older adults.

There is currently no standard definition of frailty, and previous studies have proposed various measures and evaluation methods as indicators of frailty. In the index of frailty developed by Rockwood *et al*., evaluation is based on the concept that diseases, impairments in activities of daily living (ADLs), and physical dysfunctions accumulate as people age.^3^ This is known as the accumulated deficit model. The index developed by Fried *et al*. consists of five symptoms: shrinking, weakness, exhaustion, slowness, and low activity. If a patient exhibits three or more of these symptoms, they are considered frail; if they exhibit one or two of the symptoms, they are considered pre‐frail.^4^ This is known as the phenotype model.

The Clinical Frailty Scale (CFS) developed by Rockwood *et al*. is an index of frailty and disability.^3^ The CFS comprises nine categories according to the clinical judgment of physicians and nurses, based primarily on health information of the older patient. The CFS has undergone multiple revisions and has been translated into numerous languages, some of which have been validated.^5^, ^6^, ^7^, ^8^, ^9^, ^10^ In 2021, the Japanese Geriatrics Society prepared the Japanese version of the CFS (CFS‐J), but no study has investigated its validity. Therefore, the purpose of this study was to validate the CFS‐J.

## Methods

### Setting and participants

This study was a cross‐sectional validation study. Data were obtained from a prospective cohort study of acutely hospitalized older patients. The patients aged ≥65 years who were admitted to the geriatric ward of Nagoya University Hospital were continuously recruited between June 2021 and March 2023. Patients were excluded if: (i) they were discharged within 48 h; (ii) they or their family did not provide written consent to participate; (iii) their life expectancy at admission was estimated by the attending physician to be <1 month; (iv) they were readmitted within 3 months of discharge and were enrolled in the study at the time of the previous admission; (v) they were transferred from another department; or (vi) other reasons deemed to preclude participation excluded the participant. The details are as described previously.^11^

### Data collection

Background data were obtained from clinical records and included age, sex, height, weight, and body mass index. The attending geriatrician conducted a Comprehensive Geriatric Assessment (CGA) to determine the function, cognition, nutritional status, and depression of each participant. Functional status at baseline was assessed using the Eastern Cooperative Oncology Group Performance Status (PS), which has been adopted by the World Health Organization.^12^ In addition, basic ADLs at baseline were assessed using the Barthel Index (BI).^13^ Instrumental ADLs were assessed using the Lawton and Brody scale.^14^ The nutritional status was assessed using the Mini‐Nutritional Assessment‐Short Form (MNA‐SF).^15^ Cognitive function was assessed using the Mini‐Mental State Examination (MMSE).^16^ The degree of depressive condition was assessed by the Geriatric Depression Scale‐15 (GDS‐15).^17^ Comorbidity was assessed by the Charlson Comorbidity Index, which was created to calculate predicted mortality based on comorbidities at the time of admission.^18^ Vitality in relation to ADL was assessed using the Vitality Index.^19^ A score of the CFS‐J of a participant was assessed by a single physician using the CFS‐J and referred to the CFS guidance and the CFS classification tree as needed.^20^, ^21^

### Translation of the Clinical Frailty Scale into Japanese

After obtaining permission from Rockwood and colleagues, who created the original CFS, a translation from English to Japanese was done by the Working Group to Optimize CGA Tools of The Japan Geriatrics Society (JGS). The translated version of the CFS was compared with the original CFS by members of the Academic Committee of the JGS until consensus was reached. Afterwards, a back translation was done by a professional translator. Lastly, members of the Academic Committee of the JGS participated in group discussions to compare the back translation with the original CFS. Minor discrepancies were resolved, and the final CFS‐J was in agreement with all expert reviewers.^21^

### Frailty Index based on a Comprehensive Geriatric Assessment

The FI‐CGA was used to collect information on 10 standard CGA domains, including cognition, depression, sensory system, ADL, nutrition, stairs, bladder function, bowel function, and social resources.^22^, ^23^ For each domain, “0” indicates no problem, “0.5” indicates a minor problem, and “1” indicates a major problem. Scores were summed into an impairment index ranging from 0 to 10. To construct the FI‐CGA, the sum of the impairment was further divided by the number of domains (usually 10 domains) into a score ranging from 0 to 1. If three or more domains were missing, it was treated as a missing value and excluded from the analysis. The detailed scoring criteria are presented in Table 1. With reference to previously reported cutoffs, participants were categorized as either pre‐frail (0.08< FI‐CGA <0.25) or frail (FI‐CGA ≥0.25).^24^

**Table 1:** Scoring criteria for FI and FI‐CGA

FI‐CGA domains	FI domains	Scoring methods
1. Social resource	1. Social resource	0: Not living alone
		1: Living alone
2. Cognition	2. Cognition	0: Good (MMSE ≥24)
		0.5: Fair (20 ≤ MMSE < 24)
		1: Poor (MMSE < 20)
3. Nutrition	3. Nutrition	0: Good (12 ≤ MNA‐SF ≤ 14)
		0.5: Fair (8 ≤ MNA‐SF ≤ 11)
		1: Poor (0 ≤ MNA‐SF ≤ 7)
4. Stairs	4. Stairs	0: Independent
		0.5: Needs help
		1: Unable
5. Bowel function	5. Bowel function	0: Independent
		0.5: Needs help
		1: Unable
6. Bladder function	6. Bladder function	0: Independent
		0.5: Needs help
		1: Unable
7. IADL	7. IADL	0: Independent (IADL = 8)
		1: Dependent (IADL <8)
8. Depression	8. Depression	0: Good (GDS <5)
		0.5: Fair (5 ≤ GDS < 10)
		1: Poor (GDS ≥10)
9. VI	9. VI	0: Good (VI >7)
		0.5: Fair (5 ≤ VI < 7)
		1: Poor (VI < 5)
10. Sensory system		0: No deficit in hearing or vision
		0.5: Deficit in either hearing or vision
		1: Deficit in both hearing and vision
	10. CC	0: Male ≥34 cm, female ≥33 cm
		1: Male <34 cm, female <33 cm
	11. BMI	0: BMI 18.5−25 kg/m^2^
		1: BMI <18.5 kg/m^2^ or BMI >25 kg/m^2^
	12. Feeding	0: Independent
		0.5: Needs help
		1: Unable
	13. Transfer	0: Independent
		0.5: Needs help
		1: Unable
	14. Grooming	0: Independent
		1: Unable
	15. Toilet use	0: Independent
		0.5: Needs help
		1: Unable
	16. Bathing	0: Independent
		1: Unable
	17. Mobility	0: Independent
		0.5: Needs help
		1: Unable
	18. Dressing	0: Independent
		0.5: Needs help
		1: Unable
	19. Hand grip	0: Male ≥28 kg, Female ≥18 kg
		1: Male <28 kg, Female <18 kg
	20. Polypharmacy	0: No. of drugs ≤5
		0.5: No. of drugs 5−10
		1: No. drugs >10
	21. Anemia	0: Negative (Hb ≥10 g/dL)
		1: Positive (Hb <10 g/dL)
	22. Hypoalbuminemia	0: Negative (Alb ≥4 g/dL)
		0.5: Intermediate (Alb 3.5‐4 g/dL)
		1: Positive (Alb <3.5 g/dL)
	23. Renal dysfunction	0: Negative (eGFR ≤60 mL/min/1.73 m^2^)
		0.5: Intermediate (eGFR 30‐60 mL/min/1.73 m^2^)
		1: Positive (eGFR <30 mL/min/1.73 m^2^)
	24. Hyponatremia	0: Negative (Na ≥135 mEq/L)
		0.5: Intermediate (Na 130‐135 mEq/L)
		1: Positive (Na <130 mEq/L)
	25. Liver dysfunction	0: Negative (AST ≤30 U/L, ALT ≤30 U/L)
		0.5: Intermediate (AST ≤30 U/L or ALT ≤30 U/L)
		1: Positive (AST >30 U/L, ALT >30 U/L)
	26. Diabetes mellitus	0: Negative (HbA1c <5.6%)
		0.5: Intermediate (HbA1c 5.6%‐6.5%)
		1: Positive (HbA1c >6.5%)
	27. Delirium	0: Negative
		1: Positive
	28. Fall	0: Negative
		1: Positive
	29. Decubitus ulcer	0: Negative
		1: Positive
	30. Insomnia	0: Negative
		1: Positive

Abbreviations: Alb, albumin; ALT, alanine aminotransferase; AST, aspartate aminotransferase; BMI, body mass index; CC, calf circumference; eGFR, estimated glomerular filtration rate; FI; FI‐CGA; GDS, Geriatric Depression States; Hb, hemoglobin; HbA1c, glycated hemoglobin A1c; IADL, Instrumental Activities of Daily Living; MMSE, Mini‐Mental State of Examinations; MNA‐SF, Mini‐Nutrition Assessment‐Short Form; VI, Vitality Index.

### Frailty Index

The FI is a simple yet effective tool to assess frailty, defined as a state of increased vulnerability to external stressors. The specific creation guidelines guarantee the reproducibility of the findings.^25^ Following the guidelines, the FI used in this study consists of 30 variables that measure a patient's health deficits (Table 1). To construct the CGA, the sum of the impairment score was further divided by the number of domains (usually 30 domains) into a score ranging from 0 to 1. If six or more domains were missing, it was treated as a missing value and excluded from the analysis. With reference to previously reported cutoffs, participants were categorized as either pre‐frail (0.08≤ FI <0.25) or frail (FI ≥0.25).^24^

### Validity of the Japanese version of the Clinical Frailty Scale

The concurrent criterion validity of the CFS‐J was assessed by examining the association of the CFS‐J with the FI‐CGA, FI, and PS. The correlations of the CFS‐J with other geriatric assessments (BI, Instrumental ADL [IADL], MNA‐SF, MMSE, GDS‐15, and Charlson Comorbidity Index [CCI]) were assessed by calculating the correlation coefficients of the CFS‐J with the BI, IADL, MNA‐SF, MMSE, GDS‐15, and CCI. Correlation among each category of the FI, FI‐CGA, and CFS‐J was assessed by calculating Kendall's tau. Agreement among each category of the FI, FI‐CGA, and CFS‐J was assessed by calculating the weighted kappa. The FI, FI‐CGA, and CFS‐J were categorized as robust, prefrail, or frail. The FI and FI‐CGA were categorized as mentioned above, and the CFS‐J was categorized as robust (CFS‐J = 1 or 2), pre‐frail (CFS‐J = 3 or 4), or frail (CFS‐J = 5, 6, 7, 8, or 9). We defined satisfactory validity for CFS‐J as statistically significant correlations with the existing scales. Because the CFS‐J includes a wide range of functional status between robust, pre‐frail, frail and disabled, we performed additional analyses for two specified groups: score 1–5 on the CFS‐J (group 1) and score 5–9 on the CFS‐J (group 2). CFS‐J score 5 was included in both groups (group 1 and group 2) because CFS‐J score 5 was considered as between frail and disabled status. In group 1, correlation and categorical analyses focused on frailty status were performed. In group 2, correlation and categorical analyses focused on disability status were performed. In group 2, we defined independent (≥85), nearly independent (60≤, <85), and dependent (<60) for BI, and we defined independent (5), nearly independent (6), and dependent (7–9) for CFS‐J. A receiver operating characteristic curve was drawn, and the area under the curve and CFS‐J cut‐off points were calculated in determining the frailty status diagnosed by FI (≥0.25).

### Statistical analysis

The results of the descriptive analysis are presented as *n* (%) for categorical data and as mean ± standard deviation for continuous variables. Kendall's tau for correlation was used to assess validity tests. Data were analyzed using SPSS ver. 28 (IBM Corp., Armonk, NY, USA). A two‐sided *P* value of <0.05 was considered to indicate statistical significance.

## Results

### Characteristics of the study population

Among the 223 participants analyzed in the validation study, the mean age was 84.9 ± 5.9 years (range, 67–101 years), 128 (57.4%) were women, and mean body mass index was 20.9 ± 4.0 kg/m^2^. For the frailty assessment, the CFS‐J classification ranged from 0.4% (category 1, 9) to 23.8% (category 6). When using the FI‐CGA and FI, 74.2% and 72.9% of participants were classified as frail, respectively. Other characteristics of the study population are presented in Table 2.

**Table 2:** Baseline characteristics of the study participants (*N* = 223)

Characteristics	*n* (%), mean ± SD
Age, years	84.94 ± 5.874
Female	128 (57.4)
BMI, kg/m^2^	20.88 ± 4.02
CFS‐J	5.14 ± 1.47
1	1 (0.4)
2	5 (2.2)
3	28 (12.6)
4	47 (21.1)
5	38 (17.0)
6	53 (23.8)
7	32 (14.3)
8	12 (5.4)
9	1 (0.4)
Deficit	6 (2.7)
FI (*n* = 202)	0.36 ± 0.18
FI <0.08	1 (0.5)
0.08 ≤ FI < 0.25	53 (26.2)
FI ≥ 0.25	148 (73.3)
FI‐CGA (*n* = 209)	0.42 ± 0.24
FI‐CGA < 0.08	4 (1.9)
0.08 ≤ FI‐CGA < 0.25	50 (23.9)
FI‐CGA ≥ 0.25	155 (74.2)
PS	1.75 ± 1.27
0	40 (17.9)
1	58 (26.0)
2	37 (16.6)
3	53 (23.8)
4	17 (7.6)
Deficit	18 (8.1)
Elements of geriatric assessment	
BI	72.1, 32.5
IADL	4.1 ± 3.3
MNA‐SF	8.7 ± 3.3
MMSE	20.2 ± 8.2
GDS‐15	5.8 ± 3.8
CCI	2.5 ± 1.9

Abbreviations: BI, Barthel Index; BMI, body mass index; CCI, Charlson Comorbidity Index; CFS‐J, Japanese version of the Clinical Frailty Index; CGA, Comprehensive Geriatric Assessment; GDS‐15, Geriatric Depression Scale‐15; FI, Frailty Index; IADL, Instrumental Activities of Daily Living; MMSE, Mini‐Mental State Examination; MNA‐SF, Mini‐Nutrition Assessment‐Short Form; PS, Performance Status; SD, standard deviation.

### Criterion concurrent validity

Table 3 shows the correlation coefficient among the FI, FI‐CGA, CFS‐J, and PS. CFS‐J had significant positive correlations with the FI‐CGA, the FI, and PS. All *P* values were <0.001. Table 4 shows the correlations among each categorization of the FI, FI‐CGA, and CFS‐J; each correlation had a significant positive correlation, and all *P* values were <0.001. Table S1 shows the correlation among the FI, FI‐CGA, CFS‐J, and PS, in group 1 (CFS‐J ≤5), which was focused on frailty status. In group 1, CFS‐J also had significant positive correlations with the FI‐CGA, the FI, and PS. Table S2 shows correlation and agreement among each category of the FI, FI‐CGA, and CFS‐J in group 1 (CFS‐J ≤5), which was focused on frailty status. In group 1, each correlation also had a significant positive correlation, and all *P* values were <0.001. Table S3 shows the correlation and agreement of the CFS‐J and BI in group 2 (CFS‐J ≥5), which was focused on disabled status. In group 2, the correlation and agreement of the CFS‐J and BI had a significant correlation, and all *P* values were <0.001.

**Table 3:** Correlation among the FI, FI‐CGA, CFS‐J, and PS

	FI	FI‐CGA	CFS‐J	PS
FI		0.745 (*P* < 0.001)	0.711 (*P* < 0.001)	0.616 (*P* < 0.001)
FI‐CGA			0.691 (*P* < 0.001)	0.612 (*P* < 0.001)
CFS‐J				0.669 (*P* < 0.001)
PS				

*Note*: Correlation coefficients were calculated by Kendall's tau. Abbreviations: CFS‐J, Japanese version of the Clinical Frailty Scale; FI, Frailty Index; FI‐CGA, Frailty Index based on Comprehensive Geriatric Assessment; PS, performance status.

**Table 4:** Correlation among each category of the FI, FI‐CGA, and CFS‐J

	FI‐CGA category Kendall's tau weighted kappa	CFS‐J category Kendall's tau weighted kappa
FI category	0.611 (*P* < 0.001)	0.562 (*P* < 0.001)
	0.573 (*P* < 0.001)	0.523 (*P* < 0.001)
FI‐CGA category		0.591 (*P* < 0.001)
		0.533 (*P* < 0.001)

*Note*: Correlation coefficients of category (robust, prefrail, and frail) of the FI, FI‐CGA, and CFS‐J were calculated by Kendall's tau. Degree of agreement among each category (robust, prefrail, and frail) of the FI, FI‐CGA, and CFS‐J was calculated by the weighted kappa. Abbreviations: CFS‐J, Japanese version of the Clinical Frailty Scale; FI, Frailty Index; FI‐CGA, Frailty Index based on a Comprehensive Geriatric Assessment.

Regarding the receiver operating characteristic curve for the identification of frailty (FI ≥0.25), the CFS‐J showed a good performance with an area under the curve of 0.861 (Figure S1). In addition, the CFS‐J has a sensitivity of 0.776 and a specificity of 0.852 for identifying frail (FI ≥0.25) at a cut‐off of 4/5.

### Correlation between the Japanese version of the Clinical Frailty Scale and other geriatric assessments

The CFS‐J had significant negative correlations with the BI, IADL, MNA‐SF, and MMSE and a significant positive correlation with GDS‐15 (Table 5). The correlations of the CFS‐J with BADL and IADL were higher than those with the MNA‐SF, MMSE, GDS, and CCI.

**Table 5:** Correlations of the FI, FI‐CGA, CFS‐J, and PS with geriatric assessments

	BI		IADL		MNA‐SF		MMSE		GDS‐15		CCI	
	Kendall's tau	*P*‐value	Kendall's tau	*P*‐value	Kendall's tau	*P*‐value	Kendall's tau	*P*‐value	Kendall's tau	*P*‐value	Kendall's tau	*P*‐value
FI	−0.736	<0.001	−0.664	<0.001	−0.436	<0.001	−0.498	<0.001	0.287	<0.001	0.142	0.006
FI‐CGA	−0.703	<0.001	−0.709	<0.001	−0.436	<0.001	−0.498	<0.001	0.359	<0.001	0.097	0.061
CFS‐J	−0.777	<0.001	−0.742	<0.001	−0.432	<0.001	−0.525	<0.001	0.250	<0.001	0.117	0.027
PS	−0.648	<0.001	−0.593	<0.001	−0.376	<0.001	−0.427	<0.001	0.277	<0.001	0.106	0.056

*Note*: Correlation coefficients were calculated by Kendall's tau. Abbreviations: BI, Barthel Index; CCI, Charlson Comorbidity Index; CFS‐J, Japanese version of the Clinical Frailty Index; GDS‐15, Geriatric Depression Scale‐15; FI, Frailty Index; FI‐CGA, Frailty Index based on a Comprehensive Geriatric Assessment; IADL, Instrumental Activities of Daily Living; MMSE, Mini‐Mental State Examination; MNA‐SF, Mini‐Nutrition Assessment‐Short Form; PS, Performance Status.

## Discussion

The CFS‐J demonstrated satisfactory validity for assessing frailty and disability in older hospitalized patients in Japan. There were also significant correlations with FI, FI‐CGA, PS, and various domains of the CGA, including function, cognition, depression, and nutrition, indicating that the CFS‐J is a global and comprehensive assessment of frailty and disability. Cross‐cultural research on frailty and disability in diverse populations will be facilitated by this validation of the CFS‐J.

In the 7‐point CFS study reported by Rockwood *et al*., the CFS showed a high correlation with the FI.^3^ In the present study, we compared the 9‐point CFS‐J with the FI, FI‐CGA, and PS and showed that all were significantly correlated (Table 3). Like the 7‐point CFS, the 9‐point CFS‐J may be useful as an assessment of frailty.

The correlation coefficients of the FI with the FI‐CGA, CFS‐J, and PS were 0.746, 0.713, and 0.618 (all *P* < 0.001), respectively (Table 3). The FI and FI‐CGA showed the strongest correlation because of the similarity of their endpoints. The CFS is rated on a 9‐point scale, while PS is rated on a 5‐point scale. We consider that this difference in the number of points affected the value of the correlation coefficient with FI.

In group 1, the correlation coefficients of the FI with the FI‐CGA, CFS‐J, and PS were all significant (Table S1), and the correlation and agreement among each category of the FI, FI‐CGA, and CFS‐J were all significant (Table S2). Because group 1 is the frailty‐focused group, CFS‐J is useful for measuring frailty status. In group 2, the correlation and agreement of the CFS‐J and BI correlation coefficients were all significant (Table S3). Because group 2 is the disability‐focused group, CFS is useful for measuring disabled status.

The CFS‐J was assessed as a summary score after a CGA. The CFS was used to collect information from several domains, including function, cognition, physical performance, nutrition, and depression. Our findings of significant associations between the CFS‐J and various geriatric conditions are consistent with these factors and previous studies.^26^ Among them, the BI and IADL showed strong correlations with the CFS‐J. The reason for this is that function is an important factor in the CFS classification tree.^26^ Although statistically significant associations were found between the CFS‐J and the MMSE, GDS‐15, and MNA‐SF, they were not stronger than the associations with BI and IADL. These results are similar to those of other frailty scales, including the FI, FI‐CGA, and PS, and are similar to previous studies.^5^, ^6^, ^7^, ^8^, ^9^, ^10^

There are several limitations to this study. First, an uneven distribution of the CFS‐J, with a low percentage in CFS‐J category 1, 2, and 9 limits the external validity, which may be due in part to our enrollment criteria. Second, the generalizability of our results is also limited by the single‐center and inpatient design. Validation of the CFS‐J in other settings is necessary to improve external validity.

In conclusion, the CFS‐J is a valid tool for assessing frailty and disability in older adults in Japan. It is expected that the accuracy and consistency of frailty and disability assessment in both research and clinical practice will be improved by the development of this scale.

## Disclosure statement

The authors declare no conflict of interest.

## Ethics statement

The study protocol was approved by the Ethics Committee of Nagoya University Graduate School of Medicine (approval number 2019‐0260) and adhered to the principles of the Declaration of Helsinki and its later amendments.

## Patient consent statement

Written informed consent was obtained from all study participants or a proxy.

## Supporting information


**Figure S1.** Receiver operating characteristic (ROC) analysis produced a cut‐off value for CFS‐J of 4/5 (sensitivity: 0.776; specificity: 0.852; area under the ROC curve: 0.861) for predicting frail (FI ≥0.25).

**Table S1.** Correlation among the FI, FI‐CGA, CFS‐J, and PS (group 1: CFS‐J ≤5). Correlation coefficients were calculated by Kendall's tau. CFS‐J, Japanese version of the Clinical Frailty Scale; FI, Frailty Index; FI‐CGA, Frailty Index based on Comprehensive Geriatric Assessment; PS, performance status.

**Table S2.** Correlation and agreement among each category of the FI, FI‐CGA, and CFS‐J (group 1: CFS‐J ≤5). Correlation coefficients of category (robust, prefrail, and frail) of the FI, FI‐CGA, and CFS‐J were calculated by Kendall's tau. Agreement among each category (robust, prefrail, and frail) of the FI, FI‐CGA, and CFS‐J was calculated by the weighted kappa. CFS‐J, Japanese version of the Clinical Frailty Scale; FI, Frailty Index; FI‐CGA, Frailty Index based on a Comprehensive Geriatric Assessment.

**Table S3.** Correlation and agreement of the CFS‐J and BI (group2: CFS‐J ≥5). Correlation coefficients between each score of the CFS‐J and BI and between each category (independent, nearly independent, and dependent) were calculated by Kendall's tau. Agreement between each category (same as above) of the CFS‐J and BI was calculated by the weighted kappa. BI, Barthel Index; CFS‐J, Japanese version of the Clinical Frailty Scale.

## Data Availability

Research data are not shared.
