# Supplementary material for: Validation of the Japanese version of the Clinical Frailty Scale
Source: Geriatr Gerontol Int. 2025 Feb 2;25(3):411–7. doi: 10.1111/ggi.15092 (PMC12216798; doi:10.1111/ggi.15092)
Supplement: Supplementary file 1 — Figure S1. Receiver operating characteristic (ROC) analysis produced a cut‐off value for CFS‐J of 4/5 (sensitivity: 0.776; specificity: 0.852; area under the ROC curve: 0.861) for predicting frail (FI ≥0.25). [file GGI-25-411-s004.docx]

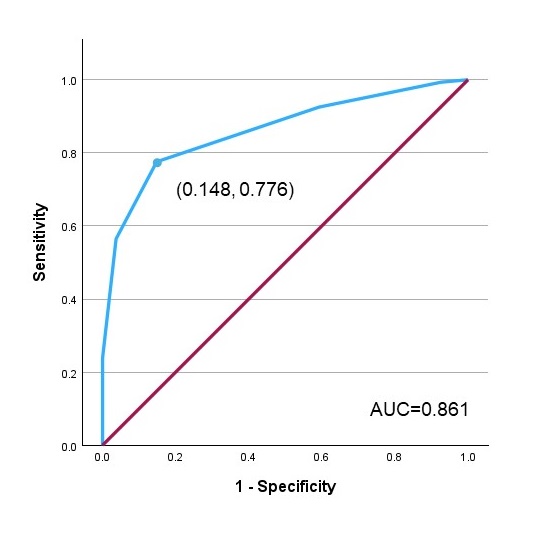


**Figure S1** Receiver operating characteristic (ROC) analysis produced a cut-off value for CFS-J of 4/5 (sensitivity 0.776; specificity 0.852; area under the ROC curve 0.861) for predicting frail (FI≥0.25).
