# Supplementary material for: Validation of the Japanese version of the Clinical Frailty Scale
Source: Geriatr Gerontol Int. 2025 Feb 2;25(3):411–7. doi: 10.1111/ggi.15092 (PMC12216798; doi:10.1111/ggi.15092)
Supplement: Supplementary file 2 — Table S1. Correlation among the FI, FI‐CGA, CFS‐J, and PS (group 1: CFS‐J ≤5). Correlation coefficients were calculated by Kendall's tau. CFS‐J, Japanese version of the Clinical Frailty Scale; FI, Frailty Index; FI‐CGA, Frailty Index based on Comprehensive Geriatric Assessment; PS, performance status. [file GGI-25-411-s003.docx]

Table S1 Correlation among the FI, FI-CGA, CFS-J, and PS (group1: CFS-J≤5)

|  | FI | FI-CGA | CFS-J | PS |
| --- | --- | --- | --- | --- |
| FI |  | 0.542 (P<0.001) | 0.443 (P<0.001) | 0.367 (P<0.001) |
| FI-CGA |  |  | 0.379 (P<0.001) | 0.342 (P<0.001) |
| CFS-J |  |  |  | 0.437 (P<0.001) |
| PS |  |  |  |  |

Note: Correlation coefficients were calculated by Kendall’s tau.

Abbreviations: CFS-J, Japanese version of the Clinical Frailty Scale; FI, Frailty Index; FI-CGA, Frailty Index based on Comprehensive Geriatric Assessment; PS, performance status.
