# Supplementary material for: Validation of the Japanese version of the Clinical Frailty Scale
Source: Geriatr Gerontol Int. 2025 Feb 2;25(3):411–7. doi: 10.1111/ggi.15092 (PMC12216798; doi:10.1111/ggi.15092)
Supplement: Supplementary file 3 — Table S2. Correlation and agreement among each category of the FI, FI‐CGA, and CFS‐J (group 1: CFS‐J ≤5). Correlation coefficients of category (robust, prefrail, and frail) of the FI, FI‐CGA, and CFS‐J were calculated by Kendall's tau. Agreement among each category (robust, prefrail, and frail) of the FI, FI‐CGA, and CFS‐J was calculated by the weighted kappa. CFS‐J, Japanese version of the Clinical Frailty Scale; FI, Frailty Index; FI‐CGA, Frailty Index based on a Comprehensive Geriatric Assessment. [file GGI-25-411-s002.docx]

Table S2 Correlation and agreement among each category of the FI, FI-CGA, and CFS-J (group1: CFS-J≤5)

|  | FI-CGA category  Kendall’s tau  Weighted kappa | CFS-J category  Kendall’s tau  Weighted kappa |
| --- | --- | --- |
| FI category | 0.504 (P<0.001)  0.460 (P<0.001) | 0.408 (P<0.001)  0.349 (P<0.001) |
| FI-CGA category |  | 0.413 (P<0.001)  0.352 (P<0.001) |

Note: Correlation coefficients of category (robust, prefrail, and frail) of the FI, FI-CGA and CFS-J were calculated by Kendall’s tau. Agreement among each category (robust, prefrail, and frail) of the FI, FI-CGA and CFS-J was calculated by the weighted kappa.

Abbreviations: CFS-J, Japanese version of the Clinical Frailty Scale; FI, Frailty Index; FI-CGA, Frailty Index based on a Comprehensive Geriatric Assessment.
