# Supplementary material for: Validation of the Japanese version of the Clinical Frailty Scale
Source: Geriatr Gerontol Int. 2025 Feb 2;25(3):411–7. doi: 10.1111/ggi.15092 (PMC12216798; doi:10.1111/ggi.15092)
Supplement: Supplementary file 4 — Table S3. Correlation and agreement of the CFS‐J and BI (group2: CFS‐J ≥5). Correlation coefficients between each score of the CFS‐J and BI and between each category (independent, nearly independent, and dependent) were calculated by Kendall's tau. Agreement between each category (same as above) of the CFS‐J and BI was calculated by the weighted kappa. BI, Barthel Index; CFS‐J, Japanese version of the Clinical Frailty Scale. [file GGI-25-411-s001.docx]

Table S3 Correlation and agreement of the CFS-J and BI (group2: CFS-J≥5)

|  | Kendall’s tau | Weighted kappa |
| --- | --- | --- |
| Score | -0.746 (P<0.001) |  |
| Category | 0.754(P<0.001) | 0.693 (P<0.001) |

Note: Correlation coefficients between each score of the CFS-J and BI and between each category (independent, nearly independent, and dependent) were calculated by Kendall’s tau. Agreement between each category (same as above) of the CFS-J and BI was calculated by the weighted kappa.

Abbreviations: BI; Barthel Index, CFS-J, Japanese version of the Clinical Frailty Scale.
